# Antidiabetic effect of gemigliptin: a systematic review and meta-analysis of randomized controlled trials with Bayesian inference through a quality management system

**DOI:** 10.1038/s41598-021-00418-z

**Published:** 2021-10-22

**Authors:** Hojin Oh, Hai Duc Nguyen, In Mo Yoon, Byung-Ryong Ahn, Min-Sun Kim

**Affiliations:** 1 College of Pharmacy and Research Institute of Life and Pharmaceutical Sciences, Sunchon National University, 255 Jungang-ro, Suncheon, Jeollanam-do 57922 Republic of Korea; 2 Unimedi Plastic Surgery Clinic, Suite 302, 3rd Floor, 833 Nonhyeon-ro, Sinsa-dong, Gangnam-gu, Seoul, 06032 Republic of Korea; 3 Korea Statistical Consulting, Suite 735, 7th Floor, 81 Sambong-ro, Jongno-gu, Seoul, 03150 Republic of Korea

**Keywords:** Endocrinology, Medical research

## Abstract

Gemigliptin is one of the latest dipeptidyl peptidase-4 inhibitors developed by LG Life Sciences. Since the early 2000s, several randomized controlled trials (RCTs) of gemigliptin have been conducted. However, no study has directly compared its antidiabetic effects through a systematic review and meta-analysis. Therefore, in this study, we performed a systematic review and meta-analysis on RCTs. In particular, a subsequent meta-analysis was performed using Bayesian inference, and an updated quality management system model was integrated throughout our study. The mean differences and 95% confidence intervals for glycated hemoglobin (HbA1c), fasting plasma glucose (FPG), homeostatic model assessment beta cell function (HOMA-β), and low-density lipoprotein (LDL) were evaluated for the efficacy outcomes of gemigliptin as compared to those of placebo and other oral antidiabetic drugs (OADs). In conclusion, we found that gemigliptin was superior to placebo and comparable to other OADs in terms of the effect on HbA1c, FPG, HOMA-β, and LDL. Further, gemigliptin was more effective than other OADs in HbA1c and HOMA-β in Bayesian inference analysis and statistically significant to other OADs in HbA1c and HOMA-β in sensitivity analysis excluding metformin. However, to confirm the results, more studies need to be analysed and the minimum clinically important difference must be applied.

## Introduction

Gemigliptin, developed by LG Life Sciences in the early 2000s and marketed as “Zemiglo” since 2012, is an oral antidiabetic agent in the dipeptidyl peptidase-4 (DPP-4) class of inhibitors^1,2^. At the time of its launch, gemigliptin was released with great anticipation. In 2019, the annual sales of gemigliptin ranked first among ethical drugs in South Korea^3^. It has been approved for new drug application in 11 countries, and many clinical trials have been performed or are in progress globally^4^. The positive results of gemigliptin trials have promoted its unique and favourable characteristics. As suggested from the suffix “gemi”, the chemical structure of gemigliptin is C_18_H_19_F_8_N_5_O_2_, and due to the ring-shaped structure of two CF_3_ molecules attached to the pyrimidino piperidine moiety, the S2 component, among the S1, S2, and S2 extensive components of DPP-4, is additionally blocked. Gemigliptin has a good inhibitory effect on DPP-4. It is a potent, selective, competitive, and long-acting DPP-4 inhibitor suitable for once daily administration with a half-life of 17–21 h, strongly acting on both alpha and beta cells in the pancreas^1,3,5^. Moreover, gemigliptin can be excreted via the liver and kidneys. Thus, it is not necessary to adjust the dose according to the degree of liver function or renal insufficiency. Patients with poor liver functions excrete the drug via the kidneys, and those with poor kidney functions mainly excrete it via the liver^6,7^. Additionally, due to no known interaction with other drugs and the small size of tablets, gemigliptin is suitable for patients with chronic diseases who take multiple drugs; moreover, as it does not interact with food, tablets can be administered regardless of the time of meal consumption^8,9^. Due to these advantages, various randomized controlled trials (RCTs) have been performed in phases II and III prior to market release, and the results have been encouraging as compared to those of its counterparts. Comparative trials have been conducted for gemigliptin and placebo in patients with type 2 diabetes^10–15^_**.**_ Also, comparative trials with oral anti-diabetic drugs (OADs), such as sitagliptin, glimepiride, metformin, linagliptin, and dapagliflozin, have been conducted in several countries^13,16–20^_**.**_ Furthermore, clinical trials have been conducted to determine the kidney protection and blood glucose variability of gemigliptin^14,17^_**.**_ Despite the many clinical results, no systematic review and meta-analysis of a direct comparison of the antidiabetic effects of gemigliptin has been published till date. Therefore, in this study, we conducted a systematic review and meta-analysis of gemigliptin by collecting RCT data. In particular, we integrated a novel and structured quality management system model from the beginning of the study to the final reporting stage along with Bayesian inference.

## Methods

This study conformed to the Preferred Reporting Items for Systematic Reviews and Meta-Analysis^21^.

### Study procedure and data quality management

**Figure 1:**
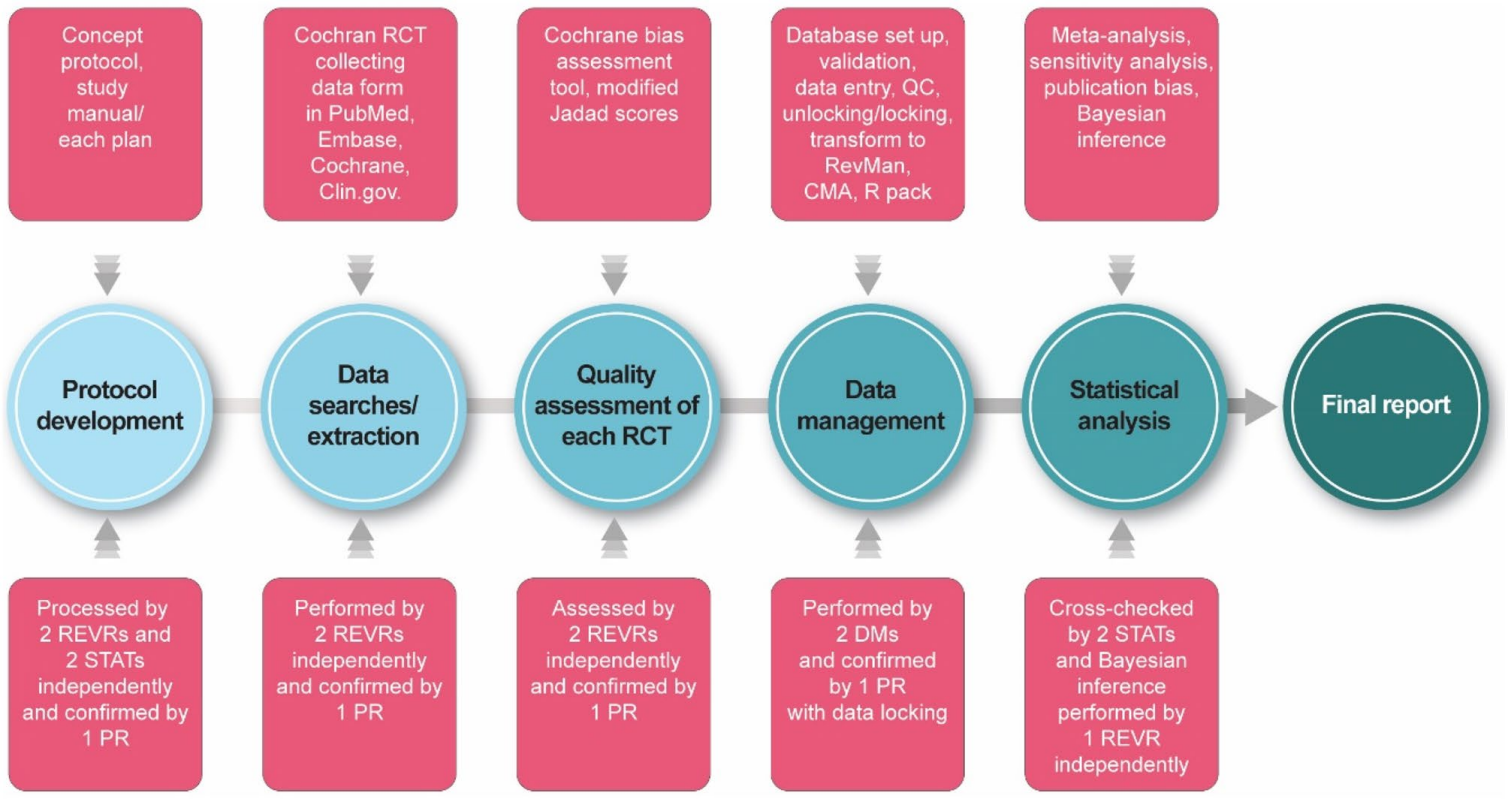
Study procedure using the quality management system.

The process of protocol development, data searches and extraction, quality assessment of each RCT, data management, and statistical analysis were the critical steps for minimizing bias and maintaining the quality of our study. The functional responsibilities for the confirmation of each step are described in Table 1. The entire study procedure through the quality management system model is depicted in Fig. 1.

**Table 1:** Functional responsibilities for the systematic review and meta-analysis.

	Project leader	Independent reviewer 1	Independent reviewer 2	Independent reviewer 3	Independent statistician 1	Independent statistician 2
Protocol development (Concept protocol, study procedure manual, each plan, etc.)	X	X	X	X	X	X
Data searches/extraction (Cochran RCT extraction form in PubMed, Embase, Cochrane, Clin.gov.)	X	X	X			
Quality assessment of each RCT (Cochrane bias assessment tool, modified Jadad scores)	X	X	X			
Data management (database set up, validation, data entry, QC, unlocking/locking, transform to RevMan, CMA, R package)	X^a^	X	X		X	X
Statistical analysis (meta-analysis, sensitivity analysis, publication bias, Bayesian inference, QC)	X			X^b^	X^c^	X^d^

*RCT* randomized controlled trial, *QC* quality control, *SA* statistical analysis, *RevMan* Review Manager, *CMA* Comprehensive Meta-Analysis.

^a^Confirmed after data locking.

^b^Performed Bayesian inference.

^c^Performed meta-analysis, sensitivity analysis, publication bias, Bayesian inference, QC.

^d^Performed meta-analysis, sensitivity analysis, publication bias, QC.

### Protocol development

The protocol development stage of our quality management system for systematic review was divided into two main stages. The first was the concept protocol phase, followed by the formal protocol development phase. In the concept protocol phase, we discussed mainly an overall outline that includes the study design, disease state, potential subjects, and inclusion/exclusion criteria. The concept protocol was required to be reviewed by the responsible personnel such as the project leader and independent reviewer 1. Following the review of the concept protocol, the writing of the formal protocol began and confirmed through final approval process. The formal protocol development phase was conducted based on the protocol criteria and Cochrane RCT data collection form, and the person in charge participated (Table 1. Functional responsibilities). Plans that were based on the protocol, such as the data management plan and statistical analysis plan, were also created in the formal protocol development phase. From the concept protocol to the final stage of the protocol development phase, the two independent reviewers and project leader continued to discuss and change the protocol amendments as needed. Each study plan within the protocol was also continuously discussed and changed until the final protocol was approved. If the major plans needed to be changed after the protocol/plans were created and the analyses commenced, they were changed according to the criteria of the mitigation/deviation plan prepared during in the protocol development stage. However, there was no major change or deviation after the analyses commenced in our research.

### Literature search

We searched PubMed, Embase, the Cochrane Central Register of Controlled Trials, Scopus, and ClinicalTrials.gov between February 2001 and February 2021. Our research team consisted of three independent reviewers, two independent statisticians, and a project leader. A literature search was initiated by two independent reviewers, and the results were finalized and approved by the project leader. We discussed the primary search strategy at the initiation meeting. Additionally, we discussed any intermediate changes and minor deviations at interim meetings according to the mitigation plan and approved them at the final meeting. The primary keywords were “LC15-0444”, “Zemiglo”, “gemigliptin”, “gemigliptin and placebo”, “gemigliptin and dipeptidyl peptidase-4”, and “Efficacy of gemigliptin”. A limit or filter applied on the search was “randomized controlled trial” or “clinical trial”. The search was limited to articles published in the English language. The Cochrane RCT data collection form was then completed by our research team according to the search strategy and inclusion/exclusion criteria set forth in the study protocol. Next, full-text, reference, and detailed reviews were conducted on the selected RCTs. In case of disagreement between the two independent reviewers during the search and review process, the final decision was made via consultation with the project leader.

### Data extraction

The standards for study extraction and classifications were as follows: (1) the general characteristics of the study (i.e., design, country, sample size, year of publication, funding, and conflict of interest), (2) target population and setting, (3) study method, (4) outcome measurement, (5) results and findings, (6) limitations and mitigation plan, and (7) conclusion from the Cochrane RCT data collection form^22^.

Inclusion criteria were as follows:

1. RCTs
2. Patients with type 2 diabetes
3. 50 mg gemigliptin (commercial dose) versus other OAD studies
4. 50 mg gemigliptin (commercial dose) versus placebo therapy studies
5. Study duration of 12 weeks or longer for primary or secondary outcomes
6. Patient age >18 years

Exclusion criteria were as follows:

1. Observation, review, case, animal, cell level, and molecular studies of gemigliptin
2. Protocol only, supplementary publications, and periodicals of gemigliptin
3. Pharmacokinetic and pharmacodynamic studies of gemigliptin
4. Gemigliptin versus its counterparts in patients with disease conditions other than type 2 diabetes

For the study protocol and search strategy prepared during the study initiation meeting, a discussion was held with two independent reviewers, two independent statisticians, and the project leader. Subsequently, the two independent reviewers were assigned the job to perform extraction and classification from the corresponding medical search engines along with the Cochrane RCT data collection form with the inclusion/exclusion criteria. A study selection related to the primary search was performed, and a subsequent decision was made by the two independent reviewers, two independent statisticians, and project leader during the final meeting. Following this, the full text and reference review, details extracted from the selected studies, and quality assessment (Cochrane Collaboration Risk of Bias Tool and Jadad score) of the final selected studies were arranged, and any alterations were managed during on-demand meetings.

### Quality assessment

For the RCTs that were ultimately selected during the data extraction process, two independent reviewers performed the quality assessment. The risk of bias for each RCT was evaluated according to the Cochrane Collaboration risk of bias tool^23^. The evaluation features random sequence generation, allocation concealment, blinding of participants and personnel, blinding of the outcome assessment, analysis of incomplete outcome data, selective reporting, and other biases. Two independent reviewers evaluated each item as “yes” or “no” for a low risk of bias or high risk of bias, respectively. If the two reviewers’ opinions differed and remained unresolved in terms of the information found, the item in question was rated as “unclear”. The rating was finalized following discussion with the project leader. We also used the modified Jadad score to check the intra-rater validity between the studies^24^. Regarding the quality of the assessment process, after an initial assessment by each independent reviewer, any discrepancy and finalization were coordinated with the consensus of the project leader for an on-demand meeting.

### Data management

The first step of data management was writing the data management plan, which included the overall study aspects, including the study structure, data validation process, data entry, database import/export, and database lock/unlock. The study structure was created in Excel based on the Cochrane RCT data collection form. The structure consisted of information on the study characteristics, participant demographics, ethnicity, withdrawals, intervention/control groups, and outcomes of the included RCTs. Subsequently, data validation was performed to ensure data completeness, consistency, and accuracy in the structure of the Cochrane RCT data collection form. The validation test was performed by an independent data manager (reviewer 1) to ensure that if the validation results were out of the data range in the structure, it would be possible to correct the query once the dummy data were input manually. Next, double entry was manually performed by two independent data managers (reviewers 1 and 2). The first and second data managers independently entered the data into the Excel structure from the Cochrane RCT data collection form. The Excel dataset was then checked for any discrepancies using the Excel function. The database lock was performed once all expected data were accounted for and all data management activities were completed. If any data change was required, the data were modified with the consensus of project leader; consensus was also obtained from each data manager to unlock the dataset, and the dataset was locked again. The final confirmation of the data lock was performed by the project leader, following which the data were read only. Following this, an independent data manager (reviewer 2) performed quality checks to ensure the correctness and completeness of the data according to the transfer specification. Finally, the same dataset was transferred to two independent statisticians for statistical analysis.

### Statistical analysis and data synthesis

Statistical analysis was performed using the summary statistics of selected RCTs. Summary statistics were generated from a dataset created through the data management process. Next, two independent statisticians applied the same dataset using Review Manager (version 5.4; Nordic Cochrane Center, Copenhagen, Denmark), performed meta-analysis, and assessed the publication bias. Sensitivity analysis was performed by two independent statisticians using Comprehensive Meta-Analysis version 3.3 (Biostat, Englewood, NJ, USA). For the final results of statistical analysis, quality control and cross-checking were performed by two independent statisticians as follows: (1) visual inspection of the dataset, (2) verification of the final result, and (3) reprogramming as needed. Any discrepancies or deviations after the quality check were discussed at the interim data meeting and reprogrammed if necessary. The final output was confirmed by consensus between the project leader and statisticians. Mean differences in glycated hemoglobin (HbA1c), fasting plasma glucose (FPG), homeostatic model assessment of beta cell function (HOMA-β), and low-density lipoprotein (LDL) and their corresponding 95% confidence intervals (CIs) were compared to baseline levels of continuous variables to determine the difference in efficacy between gemigliptin and placebo and other OADs. The missing standard deviation (SD) was determined according to the method presented in the Cochrane Handbook (Cochrane Handbook, version 6.1)^22,25^. Statistical significance was set at *P* < 0.05. Cochran’s Q and I^2^ statistics were calculated and used to evaluate the statistical heterogeneity of each selected study. We categorized the heterogeneity of studies according to the I^2^ statistics as follows: low (0–40%), moderate (30–60%), substantial (50–90%), and considerable (75–100%)^22,25^_**.**_ We also evaluated clinical heterogeneity (methodological and clinical diversity) based on each trial’s characteristics, such as study design, study duration, study papulation, the risk of bias, interventions, and outcome assessments^22,25^_**.**_ The selection of random- or fixed-effect models was made on the basis of the clinical and statistical heterogeneity. In other words, although the Cochran’s Q and I^2^ statistics can be used to categorize statistical heterogeneity, our research is in the medical field. Therefore, the variation in both the clinical heterogeneity and statistical heterogeneity among the analysed studies in our research was considered in the choice of whether to use either a random- or fixed-effect models. A funnel plot was used to graphically evaluate publication bias, and the plot was mathematically generated according to the method suggested by the Begg and Mazumdar rank test^26,27^. To validate our meta-analysis results from RevMan, Bayesian inference was used to predict the posterior median differences in HbA1c, FPG, HOMA-β, and LDL. Prior information and existing data (likelihood) were defined in the data from previous studies and the last study, respectively. The Shiny and ggsci packages in R version 3.4.1 (R Core Team, Vienna, Austria, 2014) were used to generate the plot^28^.

## Results

### Search results

**Figure 2:**
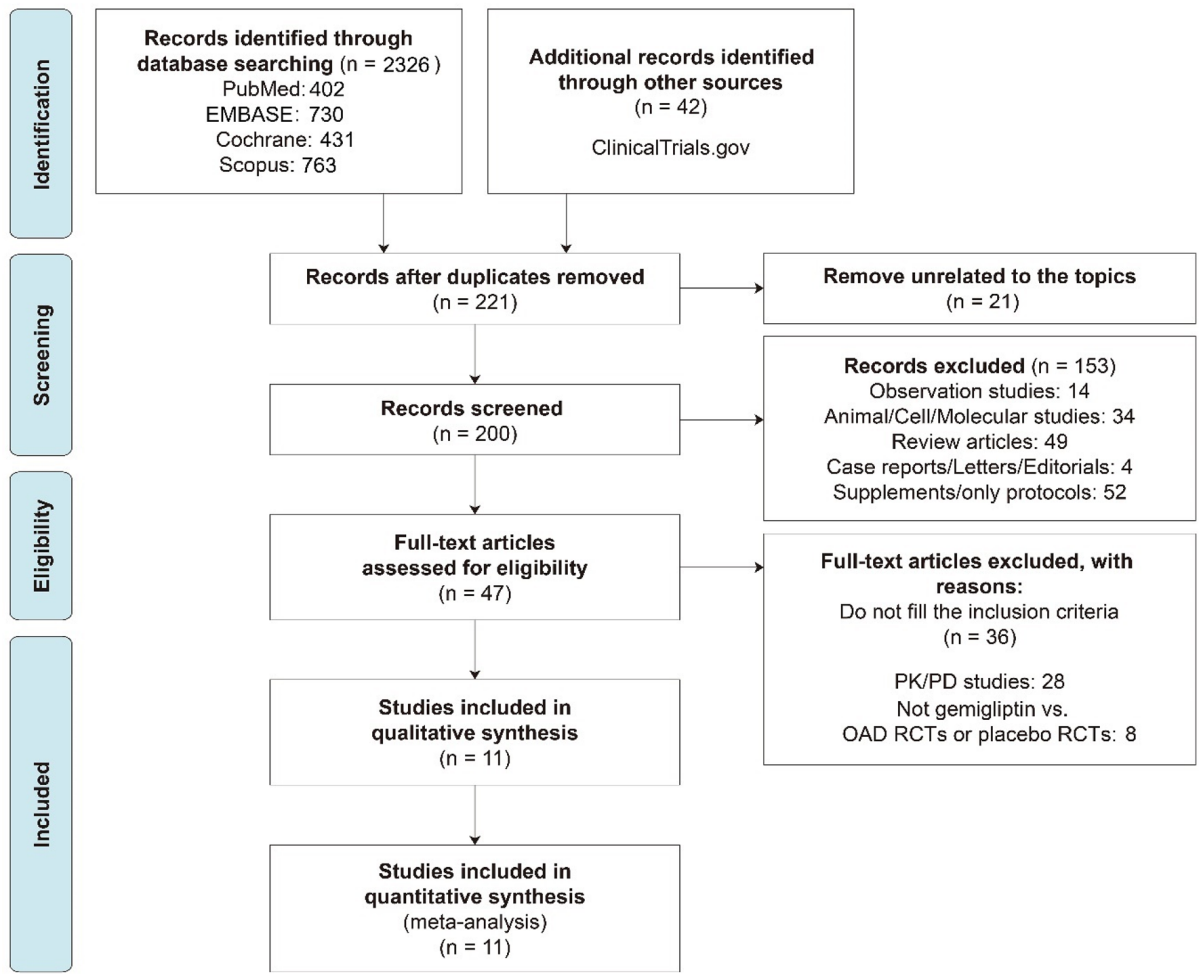
Flow chart of the studies selected and included in the meta-analysis.

Of the 2326 studies obtained via the initial primary search, 221 were evaluated for inclusion by reviewing the abstracts after excluding duplicates. The entire text of 47 studies was then evaluated by a reviewer, and 36 of the studies were excluded because they did not meet the inclusion criteria. Thus, 11 RCTs were selected for analysis. The study selection flowchart is shown in Fig. 2.

The final 11 selected studies were similar in terms of their study design, i.e., they were all randomized, active or placebo-controlled, and multicenter trials. However, the studies differed in terms of their study duration, study size or population, and intervention. Gemigliptin was compared with placebo in six studies, 100 mg sitagliptin in three studies, 5 mg linagliptin in one study, 1000 mg to 2000 mg metformin in one study, 2 mg glimepiride in one study, and 10 mg dapagliflozin in one study. The study duration of the 11 studies analysed was more than 12 weeks, according to the study inclusion criteria, which referred to the European Medicines Agency (EMA) Guideline on Clinical Investigation of Medicinal Products in the Treatment of Prevention of Diabetes Mellitus and the International Conference on Harmonization (ICH) E10 guideline for the choice of a control group in clinical trials. These guidelines state that in clinical trials in diabetes, a duration of no less than 3 months is recommended in superiority trials of new agents versus placebo, but it is also recommended that at least one active-controlled study be submitted for authorization in non-inferiority trials^29,30^. A total of 2093 subjects were included in the overall efficacy analysis, and a total of 225 subjects were withdrawn from the trials. The average age of the subjects was 55.0 years, and 57.6% of them were men. By country, five of the eleven studies were conducted in multiple nations, three were conducted in India, and two were conducted in Thailand. The study characteristics according to population, intervention, comparison, outcomes, and study design are described in Table 2. The subject demographics of each study are depicted in Table 3.

**Table 2:** Study characteristics of the 11 selected RCTs with population, intervention, comparison, outcomes, and study design.

Trial	Study design	Study size and population	Study duration	Gemigliptin dose	Primary outcome	Secondary outcome	Drop out
				Comparator dose			
Rhee 2010	RCT, Double blind, Placebo controlled, Optimal dose evaluation, Parallel, Multicenter	145 patients, Hb A1c: 7–11% Age: 18–75 years	12 w	GEM 50 mg	Change in HbA1c at 12 w	HbA1c responder rate at 12 w, Change in FPG, serum insulin, proinsulin and serum C-peptide, HOMA-β, HOMA-IR at 12 w	160 eligible participants, 145 randomized subjects, 4 excluded from efficacy analysis
				GEM 100 mg			
				GEM 200 mg			
				PBO			
Yang 2013	RCT, Double blind, Placebo controlled, Parallel, Multicenter, Multinational	182 patients, HbA1c: 7–11% Age: 18–75 years	24 w	GEM 50 mg	Change in HbA1c at 24 w	HbA1c responder rate at 24 w	GEM 50 mg = 7 Placebo = 8
				PBO			
Yoon 2017 (GUARD study)	RCT, Double blind, Placebo controlled, Parallel, 40-w extension, Multicenter	132 patients with renal impairment, HbA1c: 7–11% Age: 19–75 years	12 w	GEM 50 mg	Change in HbA1c at 12 w	HbA1c responder rate at 6 w, Change in body weight at 6 w, 12 w, Change in eGFR, UACR, FPG, glycated albumin, fructosamine, fasting serum C-peptide, HOMA- β, HOMA-IR, fasting lipid at 12 w	GEM 50 mg = 11 Placebo = 11
				PBO			
Lim 2017 (INICOM study)	RCT, Double blind, Placebo controlled, Parallel, Multicenter, Multinational	132 patients, HbA1c: 7.5–11% and FPG < 270 mg/dL Age: 19–75 years	24 w	GEM 50 mg + MET	Change in HbA1c at 24 w	HbA1c responder rate, Change FPG, fasting insulin, fasting C-peptide, HOMA-β, HOMA-IR at 24 w	GEM 50 mg + MET = 16 Placebo + MET = 17 Placebo + GEM 50 mg = 11
				PBO + MET			
				PBO + GEM 50 mg			
Ahn 2017 (TROICA study)	RCT, Double blind, Placebo controlled, Parallel, Multicenter	219 patients, HbA1c: 7–11% with glimepiride (> = 4 mg/d) and metformin (> = 1000 mg) at a stable dose Age: over 19 years	24 w	GEM 50 mg + MET + GLM	Change in HbA1c at 24 w	HbA1c responder rate, Change FPG, fasting serum insulin, fasting proinsulin and fasting C-peptide, proinsulin to insulin ratio, HOMA-IR, HOMA-β, fasting lipid variables at 24 w	GEM 50 mg + MET + GLM = 11 Placebo + MET + GLM = 5
				PBO + MET + GLM			
Cho 2020 (ZEUS II study)	RCT, Double blind, Placebo controlled, Parallel, Multicenter, Multinational	290 patients, HbA1c: 7–11% with a stable dose of insulin Age: over 19 years	24 w	GEM 50 mg + MET + INS	Change in HbA1c at 24 w	HbA1c responder rate, Change FPG, fasting C-peptide at 24 w	GEM 50 mg + MET + INS = 5 Placebo + MET + INS = 4
				PBO + MET + INS			
Rhee 2010	RCT, Double blind, Active- controlled, Parallel, Multicenter, Multinational	425 patients, Age: 18–75 years	24 w	GEM 25 mg bid	Change in HbA1c at 24 w	HbA1c responder rate, Change FPG, fasting insulin, proinsulin, HOMA-β, HOMA-IR at 24 w	GEM 25 mg BID = 5 GEM 50 mg = 7 SIT 100 mg = 9
				GEM 50 mg			
				SIT 100 mg			
Park 2017 (STABLE study)	RCT, Active controlled, Open label exploratory, Multicenter	69 patients, HbA1c: > 7.5% Age: 20–70 years	12 w	GEM 50 mg	Change in MAGE at 12 w	Change SD, MMT, CRP, nitrotyrosine, glycated albumin, fructosamine, HbA1c, FPG, fasting serum insulin, HOMA- β, HOMA-IR, LDL, HDL	GEM 50 mg = 0, SIT 100 mg = 2, GLIM 2 mg = 1
				SIT 100 mg			
				GLIM 2 mg			
Han 2018	RCT, Double blind, Placebo controlled, 40-week extension, Multicenter	132 patients with renal impairment HbA1c: 7–11% Age: 19–75 years	52 w	GEM 50 mg	Change in HbA1c at 52 w	HbA1c responder rate, change in eGFR, UACR, FPG, glycated albumin, fructosamine, fasting serum C-peptide, HOMA- β, HOMA-IR at 52 w	GEM 50 mg = 27, LIN 5 mg = 26
				LIN 5 mg			
Jung 2018	RCT, Double blind, Active controlled, Multicenter, Multinational	425 patients HbA1c: 7–11% with metformin (> = 1000 mg) at a stable dose	52 w	GEM 25 mg bid	Change in HbA1c at 52 w	HbA1c responder rate, change in FPG, serum insulin, proinsulin, serum C-peptide, HOMA-β, HOMA-IR at 52 w	GEM 25 mg = 14, GEM 50 mg = 18, SIT 100 mg = 2
				GEM 50 mg			
				SIT 100 mg			
Kwak 2020 (Stable II study)	RCT, Open blind end point, multicenter	71 patients HbA1c: 7–11% Age: 20–70 years	12 w	GEM 50 mg	Change in MAGE at 12 w	Change in MBG, SD, CV, HOMA- β, HOMA-IR, LDL, HDL	GEM 50 mg = 1, DAPA 10 mg = 3
				DAPA 10 mg			

*RCT* randomized controlled trial, *GEM* gemigliptin, *SIT* sitagliptin, *LINA* linagliptin, *MET* metformin, *GLIM* glimepiride, *DAPA* dapagliflozin, *INS* insulin, *bid* twice a day, *FPG* fasting plasma glucose, *GA* glycated albumin, *HOMA- β* homeostatic model assessment for beta cells, *HOMA-IR* homeostatic model assessment for insulin resistance, *CGM* continuous glucose monitoring, *UACR* urine albumin-to-creatinine ratio, *LDL* low-density lipoprotein, *HDL* high-density lipoprotein, *CV* coefficient of variation, *SD* standard deviation, *MMT* mixed-meal test, *CRP* C-reactive protein, *w* weeks, *eGFR* estimated glomerular filtration rate, *BMI* body mass index, *MAGE* mean amplitude of glycemic excursion.

**Table 3:** Subject demographics of the 11 selected RCTs.

Trial	Gemigliptin dose	Subject number	Age (years)	Men (%)	BMI (kg/m2)	Baseline HbA1c (%)	Baseline FPG (mg/dl)
	Comparator dose						
Rhee 2010	GEM 50 mg	35	52.4	71.4	25.14	8.24	162.9
	PBO	34	51.3	67.6	25.56	8.2	151.2
Yang 2013	GEM 50 mg	87	54	65.5	25.4	8.2	155.8
	PBO	87	52	50.6	26.7	8.3	159.7
Yoon 2017	GEM 50 mg	64	61.7	59.4	26	8.3	156.6
	PBO	66	62.3	57.6	26.5	8.4	150.6
Lim 2017	GEM 50 mg + MET	136	54.4	57.4	25.8	8.65	172.7
	PBO + MET	148	54	60.1	25.8	8.73	178.6
	PBO + GEM 50 mg	140	53.4	57.1	26.1	8.66	169.7
Ahn 2017	GEM 50 mg + MET + GLM	107	61.4	37.4	25.1	8.2	145.8
	PBO + MET + GLM	109	60.4	42.2	24.7	8.2	149.4
Cho 2020	GEM 50 mg + MET + INS	188	61.1	32.4	26.7	8.4	144.8
	PBO + MET + INS	95	59	30.5	26.8	8.4	137.3
Rhee 2013	GEM 25 mg bid	136	51.8	50	25.9	8.13	151.9
	GEM 50 mg	135	53.9	60	25.6	8.01	145
	SIT 100 mg	133	52.9	53.4	26.3	8.06	146.9
Park 2017	GEM 50 mg	24	48.9	71	26.6	9.5	183
	SIT 100 mg	21	49.6	76	25.9	9.1	181
	GLIM 2 mg	21	51.5	71	26	9.7	202
Han 2018	GEM 50 mg	48	62.2	64.6	26	8.4	162.3
	LINA 5 mg	52	62.6	63.5	26.7	8.4	150
Jung 2018	GEM 25 mg bid	58	51.6	55.2	26	8.2	152.3
	GEM 50 mg	55	54.6	60	25.5	7.9	139.3
	SIT 100 mg	44	51.8	54.5	26.6	8.08	149.26
Kwak 2020	GEM 50 mg	34	53.6	58.8	26	7.9	NA
	DAPA 10 mg	36	50.5	72.2	25.6	7.9	NA

*GEM* gemigliptin, *SIT* sitagliptin, *LINA* linagliptin, *MET* metformin, *GLIM* glimepiride, *DAPA* dapagliflozin, *INS* insulin, *bid* twice a day.

### Risk of bias assessment

**Figure 3:**
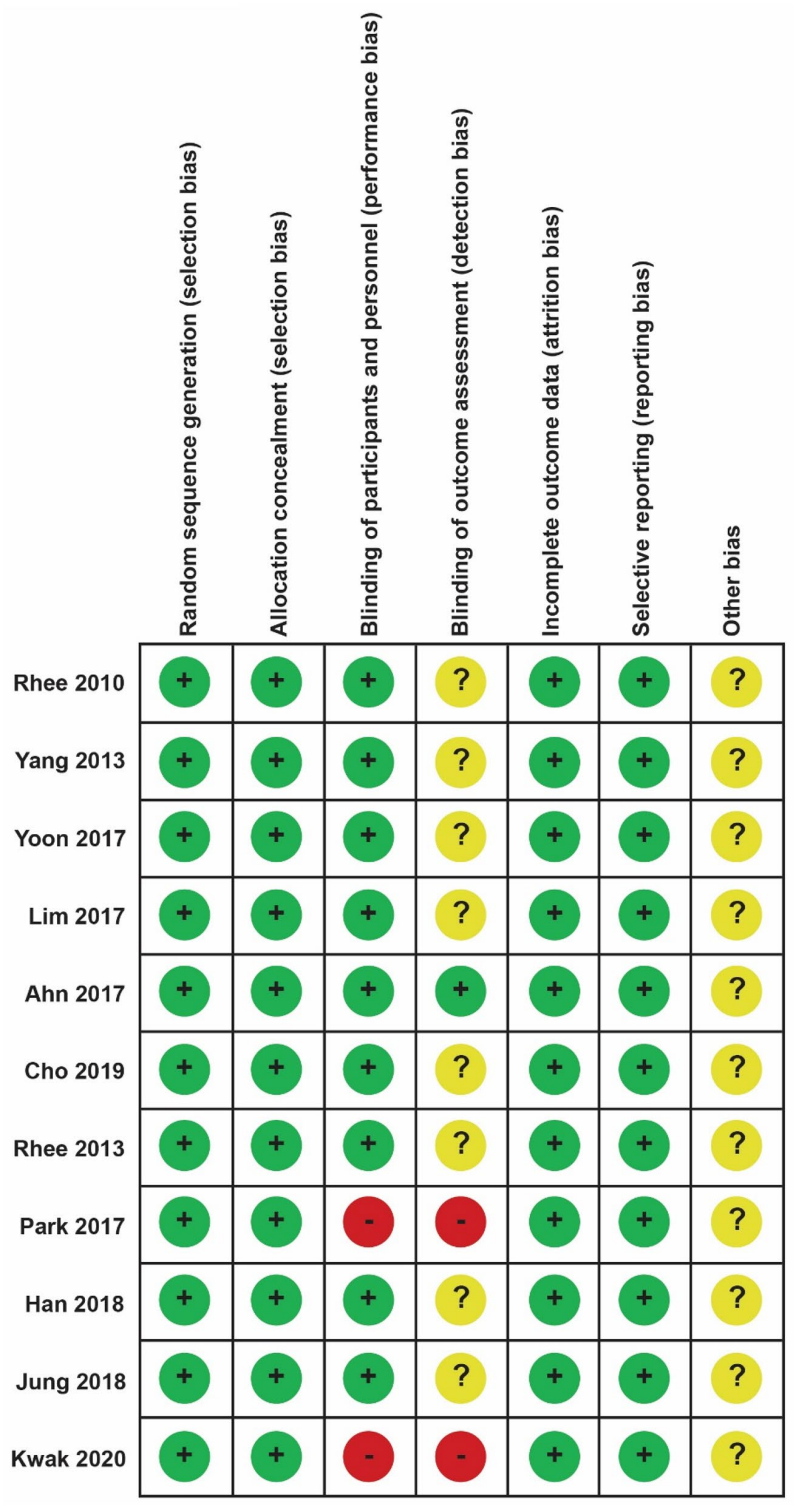
Quality assessment of the risk of bias of the eleven selected RCTs.

Among the 11 clinical trials, nine were double-blinded and two were open-label RCTs. The randomization procedure was performed using an interactive voice/web response system or a sealed envelope. Each trial was analysed appropriately in terms of the subject’ dropout or withdrawal rates. Of the 11 trials, one study was a dose-finding study in Korea^10^. Each clinical study report or the final result, including the outcome measurement, was properly reported to the authority (Ministry of Food and Drug Safety) or the institutional review board of each clinical trial institution. Other uncertain biases were assigned question marks. For the 11 selected RCTs, the risk of bias determined using the Cochrane Collaboration tool is shown in Fig. 3 and the modified Jadad scores is shown in Table 4.

**Table 4:** Modified Jadad scores of the 11 selected RCTs.

Trial	Rhee 2010	Yang 2013	Yoon2017	Lim 2017	Ahn 2017	Cho 2020	Rhee 2013	Park 2017	Han 2018	Jung 2018	Kwak 2020
Was the study described as randomized?	Y	Y	Y	Y	Y	Y	Y	Y	Y	Y	Y
Was the randomization protocol detailed and appropriate?	Y	Y	Y	Y	Y	Y	Y	Y	Y	Y	Y
Was the study described as double-blind?	Y	Y	Y	Y	Y	Y	Y	N	Y	Y	N
Was the blinding process detailed and appropriate?	Y	Y	Y	Y	Y	Y	Y	N	Y	Y	N
Did the study have a control group?	Y	Y	Y	Y	Y	Y	Y	Y	Y	Y	Y
Was the control detailed and appropriate?	Y	Y	Y	Y	Y	Y	Y	Y	Y	Y	Y
Was there an adequate exclusion criterion?	Y	Y	Y	Y	Y	Y	Y	Y	Y	Y	Y
Was the intervention used at a therapeutic dose?	N*	Y	Y	Y	Y	Y	Y	Y	Y	Y	Y
Was there a description of withdrawals and dropouts?	Y	Y	Y	Y	Y	Y	Y	Y	Y	Y	Y
Was the data clearly and adequately reported?	Y	Y	Y	Y	Y	Y	Y	Y	Y	Y	Y
Score (total = 10)	9	10	10	10	10	10	10	8	10	10	8

*Y* Yes, *N* No.

*Dose determining study for 50 mg, 100 mg, and 200 mg gemigliptin versus placebo RCT.

### Meta-analysis results

**Figure 4:**
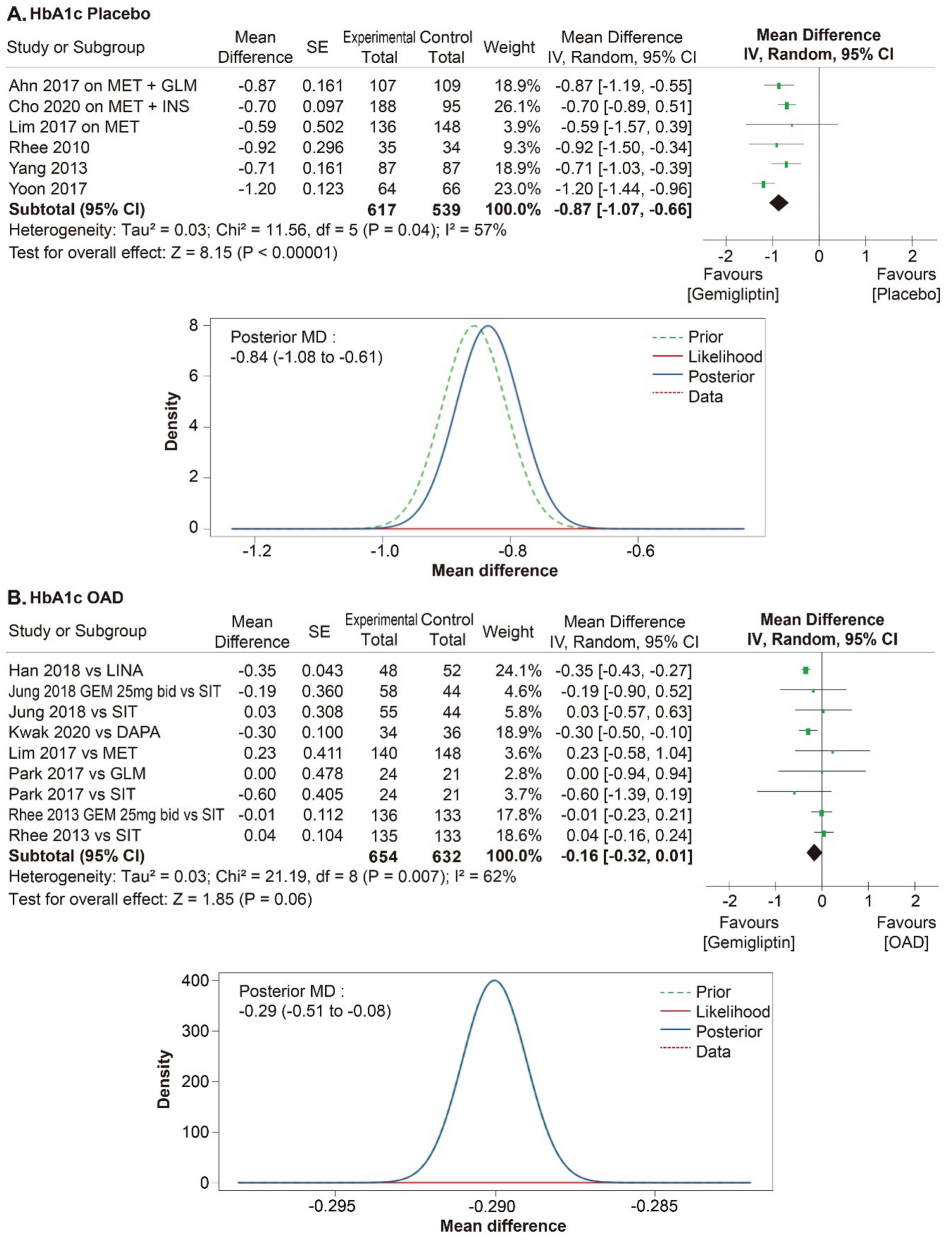
Forest plot of RCTs for the effect of gemigliptin on HbA1c.

**Figure 5:**
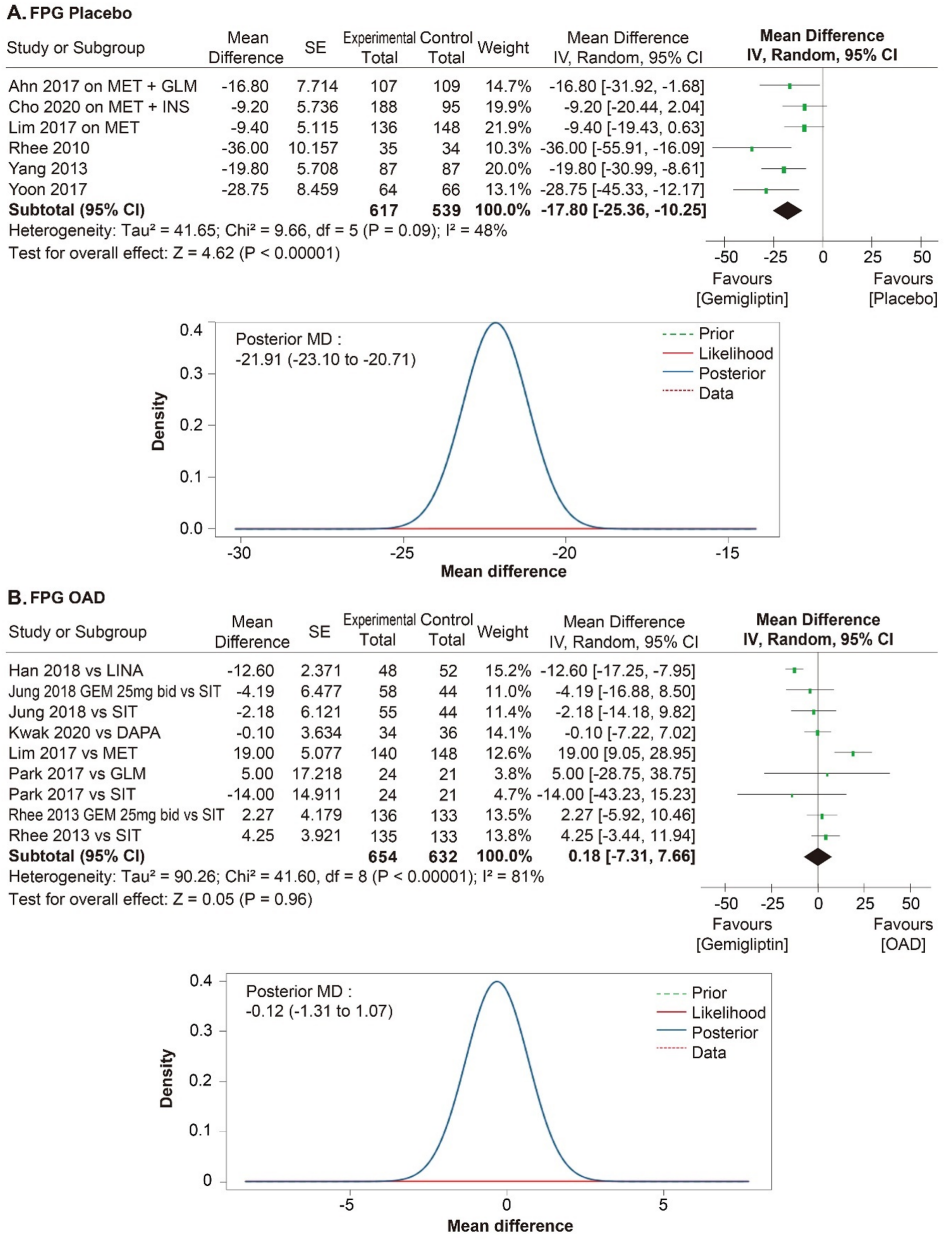
Forrest plot of RCTs for the effect of gemigliptin on FPG.

**Figure 6:**
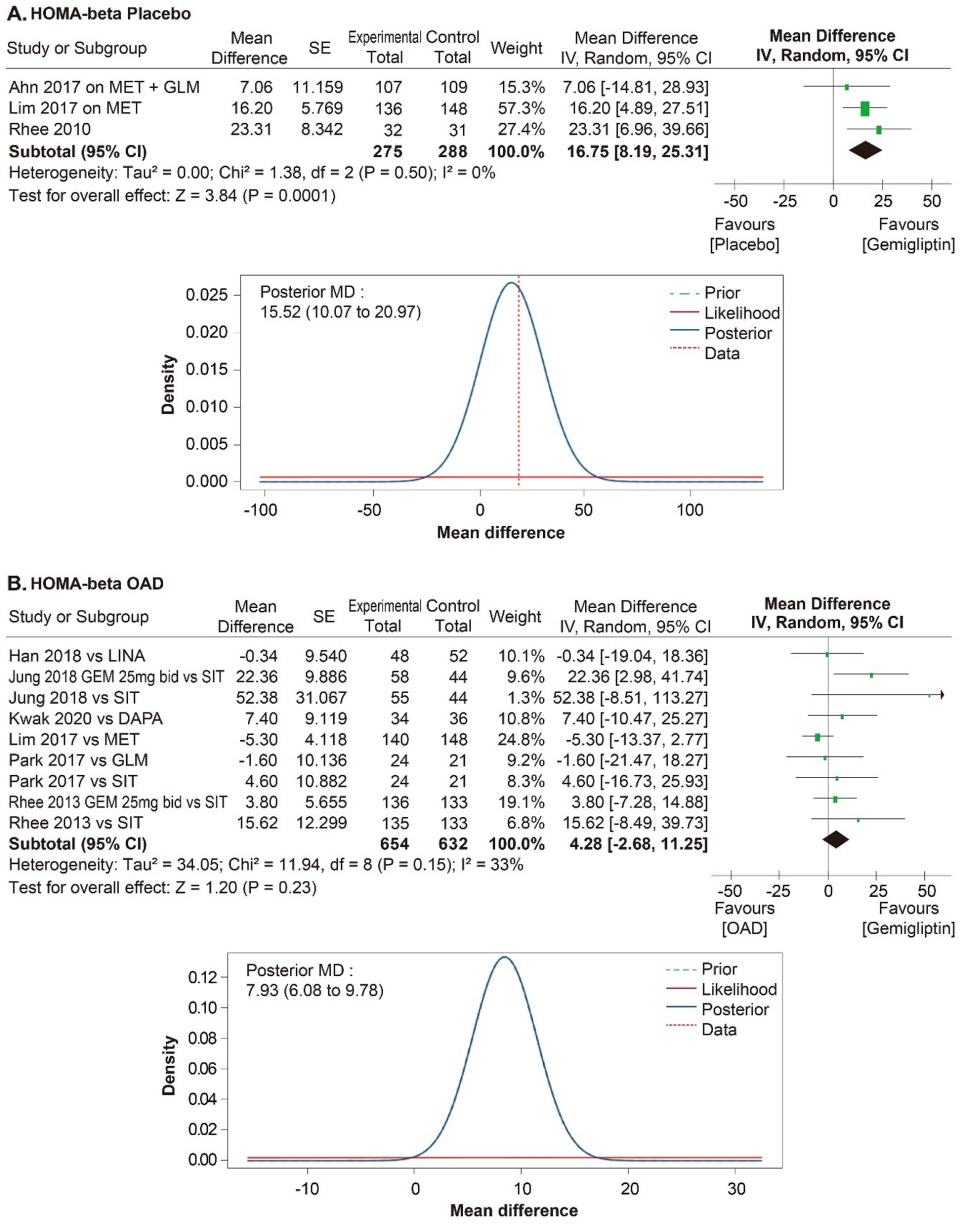
Forest plot of RCTs for the effect of gemigliptin on HOMA-β.

**Figure 7:**
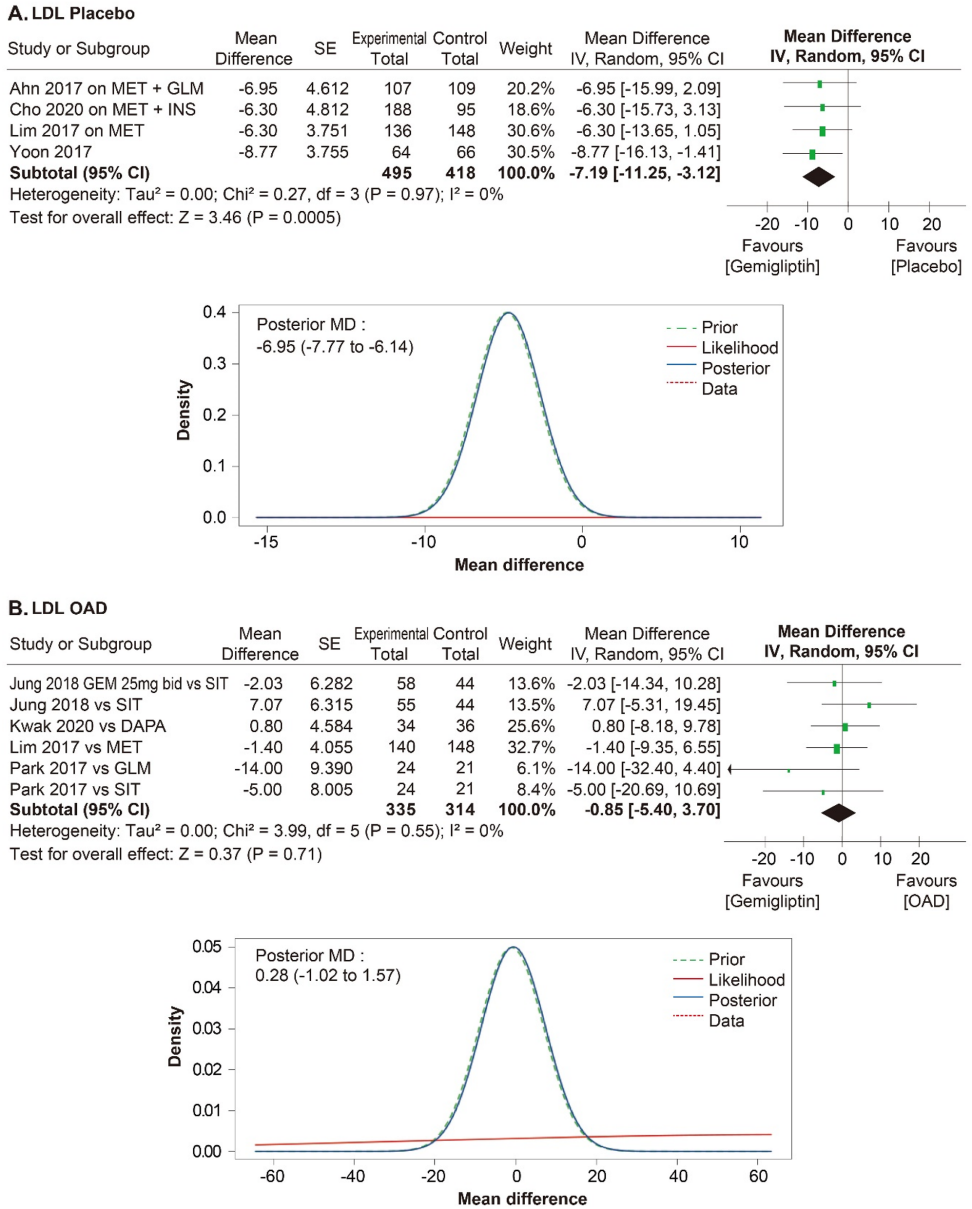
Forest plot of RCTs for the effect of gemigliptin on LDL.

A meta-analysis was performed in 11 RCTs to evaluate the efficacy of gemigliptin. The effect size of gemigliptin and placebo for HbA1c was − 0.87% (95% CI − 1.07% to − 0.66%, statistical heterogeneity, Q = 11.56, I^2^ = 57%; Fig. 4), and gemigliptin showed a significant improvement. The effect size of gemigliptin and placebo for FPG was − 17.80 mg/dL (95% CI  − 25.36 mg/dL to − 10.25 mg/dL, statistical heterogeneity, Q = 9.66, I^2^ = 48%; Fig. 5), and gemigliptin effects significantly improved. The effect size of gemigliptin and placebo for HOMA-β was 16.75 (95% CI 8.19 to 25.31, statistical heterogeneity, Q = 1.38, I^2^ = 0%; Fig. 6), and gemigliptin showed a significant improvement. The effect size of gemigliptin and placebo for LDL was − 7.19 mg/dL (CI 95% − 11.25 mg/dL to − 3.12 mg/dL, statistical heterogeneity, Q = 0.27, I^2^ = 0%; Fig. 7), and gemigliptin effects significantly improved. The effect of gemigliptin and placebo on HbA1c, FPG, HOMA-β, and LDL was in agreement with the posterior median effect size of Bayesian inference.

The effect size of gemigliptin and OADs for HbA1c was − 0.16% (CI 95%: − 0.32% to 0.01%, statistical heterogeneity, Q = 21.19, I^2^ = 62%; Fig. 4), and the effect was not significantly different between the two groups. The effect size of gemigliptin and OADs for FPG was 0.18 mg/dL (95% CI  − 7.31 mg/dL to 7.66 mg/dL, statistical heterogeneity, Q = 41.60, I^2^ = 81%; Fig. 5), and the effect was not significantly different between the two groups. The effect size of gemigliptin and OADs for HOMA-β was 4.28 (CI 95%  − 2.68 to 11.25, statistical heterogeneity, Q = 11.94, I^2^ = 33%; Fig. 6), and the effect was not significantly different between the two groups. The effect size of gemigliptin and OADs for LDL was − 0.85 mg/dL (CI 95% − 5.40 mg/dL to 3.70 mg/dL, statistical heterogeneity, Q = 3.99, I^2^ = 0%; Fig. 6), and the effect was not significantly different between the two groups. The effects of gemigliptin and OADs on FPG and LDL were in agreement with the posterior median effect size of Bayesian inference. The effect of gemigliptin with the posterior median effect size of Bayesian inference for HbA1c versus OAD was − 0.29% (CI 95% − 0.51% to − 0.08%; Fig. 3), suggesting that gemigliptin was more effective than OADs. The effect of gemigliptin with the posterior median effect size of Bayesian inference for HOMA-β versus OAD was 7.93 (CI 95% 6.08 to 9.78; Fig. 5), suggesting that gemigliptin was more effective than OADs.

### Sensitivity analysis

**Figure 8:**
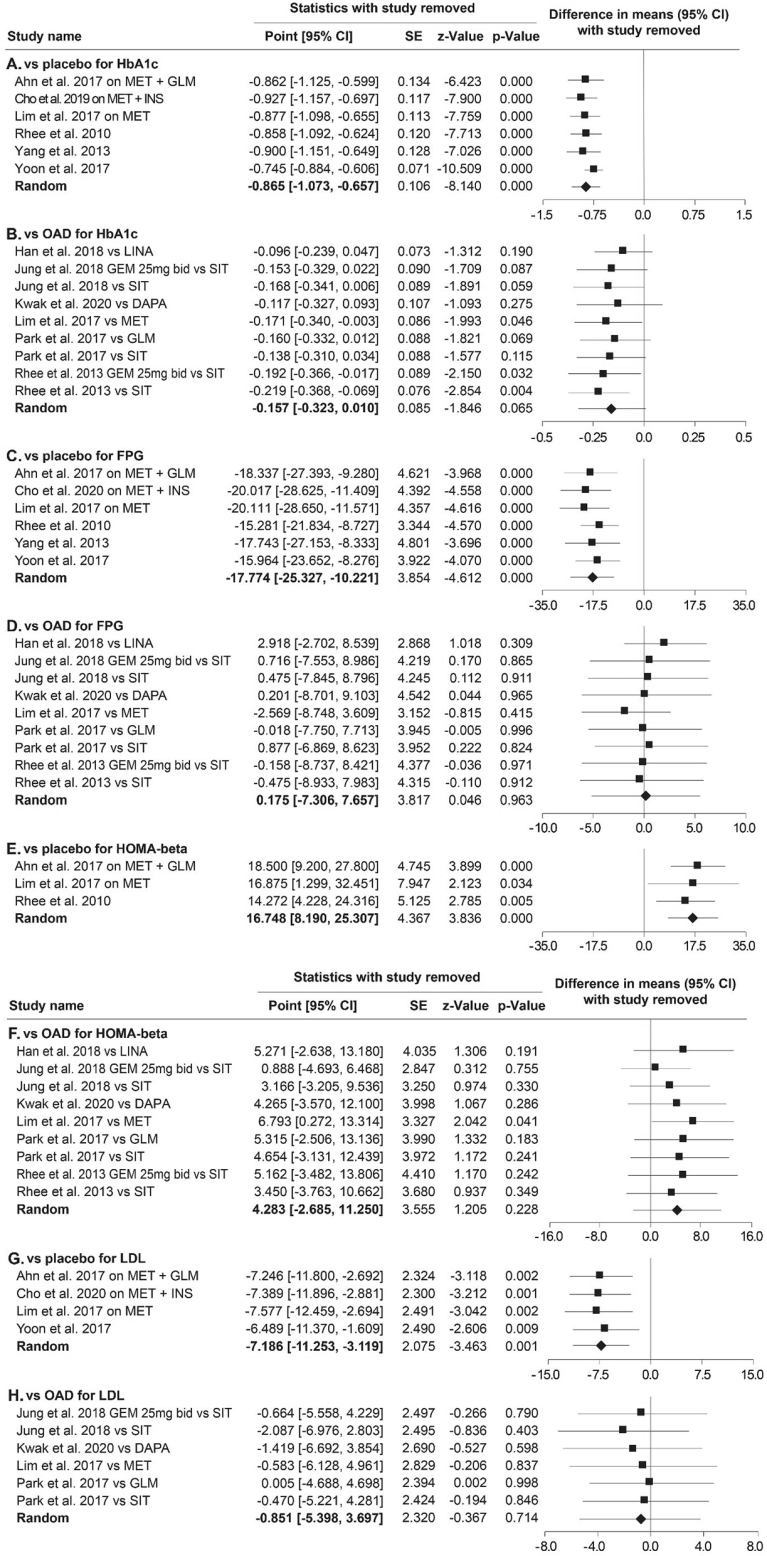
Sensitivity analysis of the studies examined for HbA1c, FPG, HOMA-β, and LDL.

Sensitivity analysis was conducted on the effect size for HbA1c, FPG, HOMA- β, and LDL by removing one study at a time. The effect size for HbA1c versus OADs was significantly different after the removal of Lim et al*.* 2017 versus metformin^13^ (mean difference (MD): − 0.17%, CI − 0.34% to − 0.003%, *p* = 0.046) and of Rhee et al. 2013 versus sitagliptin^16^ (MD: − 0.22%, CI –0.368% to − 0.069%, *p* = 0.004). The effect size for HOMA-β versus OADs was significantly different after the removal of Lim et al*.* 2017 versus metformin^13^ (MD: 6.79, CI 0.272 to 13.314, *p* = 0.041). The results were analysed using Comprehensive Meta-Analysis Software (version 3.3), and they are shown in Fig. 8.

### Publication bias

**Figure 9:**
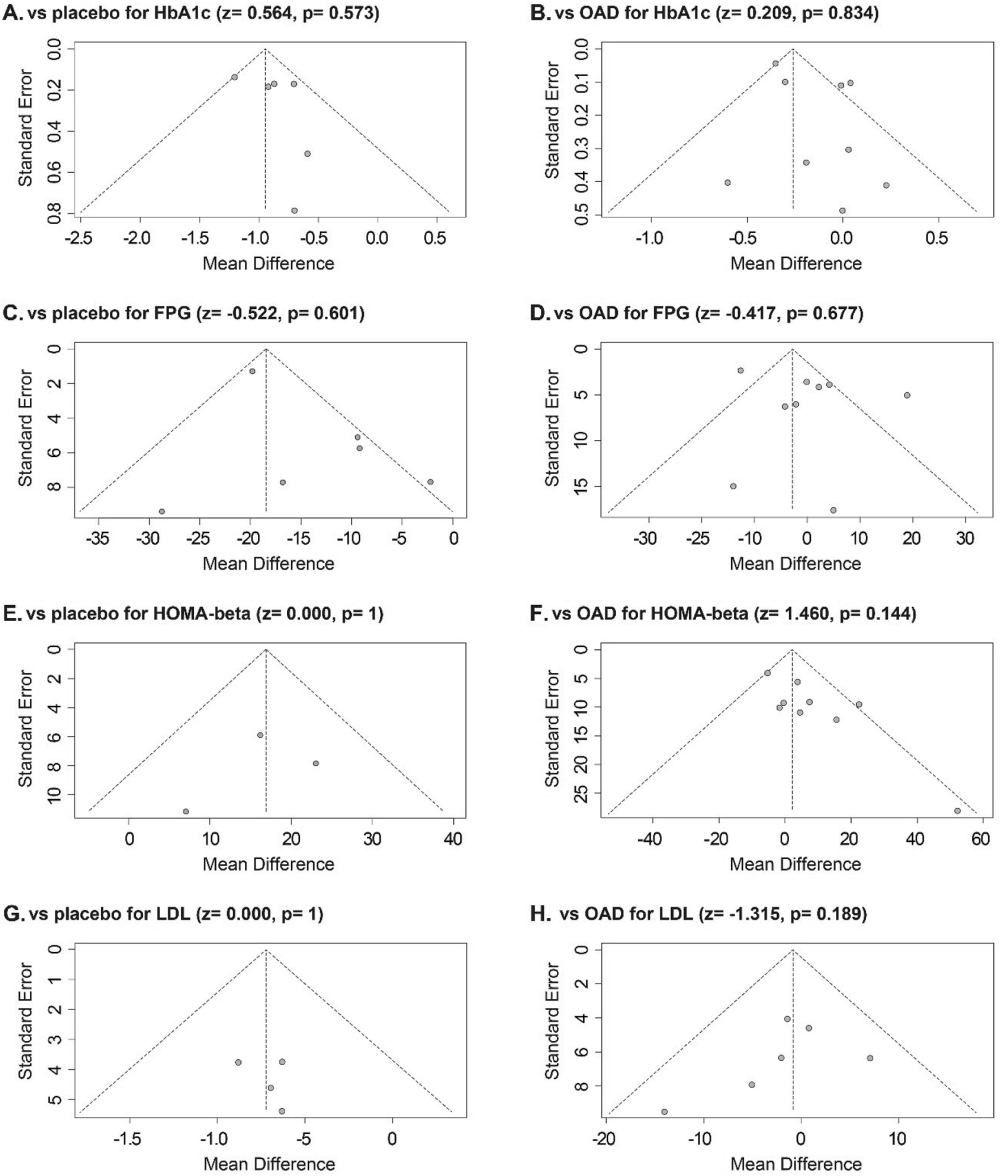
Funnel plots of the MDs versus the standard error of the MDs for HbA1c, FPG, HOMA-β, and LDL.

The Begg test was performed to assess mathematical publication bias for the meta-analysis. The results demonstrated that no significant publication bias existed for gemigliptin versus placebo as well as OADs for HbA1c, FPG, HOMA-β, and LDL. Additionally, to visualize the publication bias graphically, funnel plots were generated via Review Manager (version 5.4, Fig. 9) using Begg’s numerical value.

## Discussion

A systematic review and meta-analysis with Bayesian inference for gemigliptin, one of the latest DPP-4 inhibitors with many pharmacological advantages, was performed with an upgraded quality management system. Our review, which included a novel systematic quality management system model for analysis, is different from other systematic reviews. To maintain the quality of the review the five main steps of protocol development, data search and extraction, quality assessment of each RCT, data management, and statistical analysis, a cross-checking system was performed from the first to the final step of each stage. Particularly, in the system model structure, from the Cochrane RCT data collection form to the data management stage, two independent reviewers performed cross-checking for quality control of the dataset. Notably, statistical analysis was performed using RevMan, CMA, and R-pack, and the results were cross-checked by two independent statisticians. Additionally, another reviewer performed Bayesian inference to validate the results obtained, and the differences were reported. In comparison to the conventional method, the Bayesian approach has several practical advantages. First, the Bayesian approach allows the explicit integration of prior knowledge with new empirical evidence. Second, the use of the Bayesian approach can avoid the possible misinterpretation of *p*-values produced by megatrial populations or a summary statistic with a natural, clinically relevant interpretation—the likelihood that the research hypothesis is true given the observations. Therefore, rather than the single null magnitude to which the *p*-value refers, this posterior probability estimates the likelihood of various magnitudes of treatment effects. Taken together, the advantages indicate that the Bayesian approach is useful in clinical megatrial design, analysis, and interpretation^28^. On examination of the results obtained using this differentiated method, gemigliptin appeared to be superior to other OADs in its effect on HbA1c and HOMA-β. Although the difference was not significant in HbA1c and HOMA-β, Bayesian inference showed that gemigliptin favoured differences as compared to other OADs. Additionally, in the sensitivity analysis, after excluding the metformin study (Lim et al. 2017)^13^, a significant difference was observed in HbA1c and HOMA-β, which was in accordance with the Bayesian inference results. However, to the best of our knowledge, statistical and clinical significance should be interpreted differently. Furthermore, it is important for clinicians to determine both statistical and clinical significance in the medical field. Therefore, we applied the concept of the minimum clinically important difference (MCID) to the outcomes of our research. It has been reported in the literature that the MCID HbA1c is in the range of 0.3–0.4%. In particular, the noninferiority margin of the MCID cut-off is set at approximately 0.4% when a drug is compared to placebo in the NDA-level literature on vildagliptin, linagliptin, and saxagliptin^31–33^. According to the Center for Drug Evaluation and Research (CDER) guidance for industry on developing drugs and therapeutic biologics for diabetes mellitus, the MCID for HbA1c is 0.3%^34,35^. Therefore, from these studies, it might be said that the difference in the effect of gemigliptin on HbA1c when compared with that of placebo in our study is an MCID, whereas this difference is not only statistically nonsignificance but also not an MCID when compared with other OADs. Moreover, the MD in the effect size of − 0.171% for HbA1c obtained by removing Lim et al. 2017 (excluding vs. metformin study) in the sensitivity analysis of our study was not an MCID, although it was statistically significant. For FPG, we used 0.5 mmol/L (= 9 mg/dL) according to Viguiliouk et al. and Johnston et al^34,36^. Therefore, based on these studies, it might be said that the difference in the effect of gemigliptin on FPG is an MCID when compared to that of placebo in our study, whereas this difference is not only statistically nonsignificant but also not an MCID when compared with other OADs. For HOMA-β, we used 0.5 mmol/L for fasting glucose and 5 pmol/L for fasting insulin according to Viguiliouk et al. and Johnston *et al*^34,36^, and the value of the MCID obtained by the equation for HOMA-β, [360 × fasting insulin(μU/mL)/fasting glucose(mg/dL)-63]^37,38^ was 4.8. Thus, it might be said that the difference in the effect of gemigliptin on HOMA-β is an MCID when compared to that of placebo in our study, whereas this difference is not only statistically nonsignificant but also not an MCID when compared with other OADs. However, the 6.793 HOMA-β obtained with the removal of the study of Lim et al. (excluding vs. metformin study) in the sensitivity analysis of our study might indicate an MCID. For LDL, we used 0.1 mmol/L (= 3.86 mg/dL) for blood lipids according to Viguiliouk et al.and Johnston *et al*^34,36^. Thus, it might be said that the difference in the effect of gemigliptin on LDL is an MCID when compared to that of placebo in our study, whereas this difference is not only statistically nonsignificant but also not an MCID when compared with other OADs.

Looking at the reasons for such competitive results. In terms of structure, as compared to sitagliptin, gemigliptin is a compound designed to bind more tightly not only to the S2 subunits but also to the S1 and S2 pockets, which are DPP-4 active sites^1,3^. The binding rate of gemigliptin to the DPP-4 enzyme was similar to that of sitagliptin, but gemigliptin showed an excellent sustained effect, as the dissociation rate was five times slower. Looking at the inhibitory concentration (IC50) value, gemigliptin exhibited superior DPP-4 inhibition at 6.31 nM as compared to sitagliptin (19 nM), vildagliptin (62 nM), saxagliptin (50 nM), and alogliptin (24 nM). Higher the binding capacities of DPP-8, DPP-9, and fibroblast activation protein (FAP), which are structurally and functionally similar to DPP-4, higher the risk of drug side effects; however, drugs with a high selectivity for DPP-4 are safe. Gemigliptin has a high selectivity for DPP-4; moreover, its IC50 values for DPP-8, DPP-9, and FAP inhibition were 9565, 3412, and 22,458 times higher, respectively, than that of DPP-4. In the case of sitagliptin, the IC50 values for DPP-8, DPP-9, and FPP were 2600 times or more, 5500 times or more, and 5500 times or more, respectively, as compared to that of DPP-4. However, vildagliptin, saxagliptin, and linagliptin showed a relatively low selectivity^39,40^. Although the clinical difference due to DPP-4 selectivity was not remarkable, side effects such as hair loss, anemia, and thrombocytopenia were sometimes observed in animals. For long-term administration, drugs with a high DPP-4 selectivity are recommended.

In terms of in vitro drug-drug interactions, gemigliptin was not an inhibitor or inducer of the CYP450 family. Additionally, it did not induce *p*-glycoprotein (p-gp), but mildly inhibited p-gp at high concentrations. Even on combination with other antidiabetic agents, such as metformin, pioglitazone, and glimepiride, and antihypertensive and lipid-lowering agents, such as irbesartan and rosuvastatin, no change was observed in its pharmacokinetic parameters. However, coadministration with ketoconazole, a potent inhibitor of CYP3A4, caused a moderate increase in the total active moiety of gemigliptin by 1.9-fold with no dosage adjustment^8^. In terms of food-drug interactions, no difference was observed in Cmax and AUClast between the fasting and fed states, and gemigliptin can be administered regardless of food consumption^9^. A lack of interaction between drugs and food can be an advantage for patients who take multiple drugs with complex dosing regimens for chronic diseases such as heart disease, stroke, renal disease, and liver failure.

In terms of drug metabolism, gemigliptin has an excellent property of dual excretion, in which the drug is excreted via both the liver and kidneys. The drug is excreted via the enterohepatic system in patients with renal failure whereas it is excreted via the kidneys in patients with liver failure. Due to these complementary effects, dose adjustment is not required in patients with renal or hepatic impairments. Considering that sitagliptin, vildagliptin, and saxagliptin are mainly excreted via the kidneys and linagliptin is excreted mainly via the liver, dual excretion is a unique advantage of gemigliptin^6,7^.

Approximately 10–20% of patients with type 2 diabetes have renal diseases at the time of diagnosis. It is important to control and protect renal function due to the strong association between type 2 diabetes and renal diseases^41^. Additionally, in patients with a urine albumin/creatine ratio of 30–300 mg/g, treatment is required because cardiovascular mortality increases 2–3 times even without symptoms^42^. In the “GUARD, Yoon 2017” study, the main clinical trial was conducted in patients with renal impairment. Gemigliptin and placebo for 12 weeks were compared in type 2 diabetes patients with moderate or severe renal dysfunction. After 12 weeks, the placebo was replaced with linagliptin for up to 40 weeks. The results showed that HbA1c decreased by approximately 1.2% in the treatment group compared with the placebo group, and the urine albumin-to-creatinine ratio was significantly reduced after 12 weeks. Although not statistically significant, gemigliptin showed an enhanced blood-lowering effect as compared to linagliptin, regardless of the glomerular filtration rate^14^. Additionally, in an in vitro study on renal-protective effects, gemigliptin reduced albuminuria and lowered oxidative stress in the kidneys, thus, suggesting a potential renal-protective effect by reducing podocyte damage and renal fibrosis^43,44^.

On the other hand, the “STABLE, Park 2017” study investigated the recurrence of major cardiovascular diseases in patients with acute myocardial infarction with variability in blood glucose levels. To examine the fluctuations in blood sugar, which is an important factor that causes cardiovascular diseases and diabetes complications, the continuous glucose monitoring system method was used to view the mean amplitude of glycemic excursion (MAGE) and SD. In patients with type 2 diabetes with an HbA1c value of 7.5% or more, 50 mg gemigliptin, 100 mg sitagliptin, and 2 mg glimepiride were administered in combination with metformin. The results of the study indicated that gemigliptin showed a rapid blood glucose-lowering effect similar to glimepiride. Gemigliptin not only reduced the blood sugar levels but also reduced the blood glucose fluctuations. Gemigliptin significantly decreased both the MAGE and SD as compared to glimepiride. Furthermore, the SD significantly decreased with gemigliptin compared with sitagliptin^17^.

In the “INICOM, Lim 2017” study, to test the combination therapy of gemigliptin and metformin in early diabetic patients with a prevalence period of approximately 4 years, HbA1c levels were analysed in patients with an HbA1c value greater than 7.5% to enable the administration of the initial combination of 50 mg gemigliptin + metformin. The effects were compared in three groups: the combination therapy, 50 mg gemigliptin monotherapy, and metformin monotherapy groups. The results of the study showed that in patients who received the combination therapy, HbA1c decreased by 2.06% and a statistically significant reduction was observed in the combination therapy group compared with the monotherapy groups. Additionally, this study was different from other DPP-4 inhibitor studies that compared 2000 mg metformin versus a combination of gemigliptin and 1700 mg metformin. Notably, in the INICOM study, the dose of metformin decreased in the gemigliptin-administered group, but the effects were similar to those of other DPP-4 inhibitor + metformin groups^13^.

In terms of the effect of ethnicity on gemigliptin, six of the 11 RCTs included in our study were multinational clinical trials, and no differences were observed in gemigliptin effects between races and countries in all trials. Moreover, multinational clinical trials have been completed or are currently in progress in Russia, Mexico, and Thailand, and additional data on race are expected to be collected^39^.

However, our study has several limitations. Even though the 11 selected RCTs were large-scale multicenter clinical trials, the total number of participants was still small N to confirm the results. Although the number of participants included in our study is small, it is encouraging that gemigliptin is a drug that has been heavily involved by more than 100 multinational clinical trial institutions and practitioners in the 11 RCTs. The second limitation is that although metformin, glimepiride, dapagliflozin, and two DPP-4 inhibitors, sitagliptin and linagliptin, were included in our study, OADs from several other families should also be included. Among the several different DPP-4 inhibitors currently approved in the world, only sitagliptin and linagliptin have been compared with gemigliptin, which is a limitation of our study. To confirm the results of our research, future RCTs should compare gemigliptin with other DPP-4 inhibitors and antidiabetic agents. The third limitation is the heterogeneity of the studies analysed in our research. We acknowledge that each series of drugs in DPP-4 inhibitors, biguanides, SGLT2 inhibitors, and sulfonylureas might lead to heterogeneity in our study results because the mechanism of action (MOA) of these drugs is different. However, each NDA-level clinical trial we selected and analysed was an RCT performed within strict Good Clinical Practice (GCP) procedure. Among the 11 clinical trials, nine were double-blinded, and two were open-label RCTs. Each trial attempted to minimize bias and confounding variables regarding its study design and procedure. In addition, DPP-4 inhibitors, biguanides, SGLT2 inhibitors, and sulfonylureas drugs, even if they involve other MOAs, mainly serve to lower HbA1c and FPG and have other antidiabetic effects for the purpose of favouring the same. Our study compared gemigliptin to other OADs as a group within the framework considering antidiabetic therapy. In this regard, it can be very difficult to distinguish whether heterogeneity is due to clinical or methodological variability. In particular, the judgement should not be based solely on statistical tests for heterogeneity. The judgement of heterogenicity and then the choice of whether to use random- or fixed-effect models in meta-analysis, especially in the medical field, should be done first on the basis of clinical heterogeneity then on the basis of statistical heterogeneity. To address this point, we chose to use the random effect model due to possible clinical heterogeneity. The fourth limitation is that the interpretation of the MCID was necessary, even if the sensitivity analysis of our research showed that several outcomes (HbA1c, HOMA-β) were statistically significance of gemigliptin compared to OADs. Thus, we established MCIDs for each of our outcomes.

In conclusion, gemigliptin has many pharmacological advantages, namely a favourable tendency for cardiovascular disease and renal protection in clinical practice, an excellent effect as compared to other OADs, and a lower dose when combined with metformin. Gemigliptin appears to be superior to placebo and comparable to OADs. In particular, we observed an improved effectiveness of gemigliptin on HbA1c and HOMA-β compared to OADs in Bayesian inference and a significant improvement in sensitivity analysis after removing the metformin study. However, to confirm the results, it is necessary to gather and analysed more gemigliptin RCTs. In addition, clinicians should judge the results using MCIDs for the outcomes in practice. Our study emphasizes the strong position of gemigliptin as a current leader in oral antidiabetic therapy for type 2 diabetes. The strength of our study is that we used a novel quality management system model for systematic review and Bayesian inference with a direct comparison of meta-analysis. We believe that the quality management system and Bayesian inference applied in our study will be a prototype that can ensure quality in large-scale systematic reviews to be performed in the future.
